# Comparative Evaluation of Effectiveness of Oxygen-Releasing Gel With Scaling and Root Planing (SRP) With That of Scaling and Root Planing Alone in the Management of Chronic Periodontitis Patients: Study Protocol for Cross-Sectional Survey

**DOI:** 10.2196/75795

**Published:** 2026-03-02

**Authors:** Geeta Bhandari, Priyanka Jaiswal, Shweta Bhagat, Sakshi Kotecha

**Affiliations:** 1 Sharad Pawar Dental College and Hospital, Datta Meghe Institute of Higher Education and Research (DMIHER(DU)), Sawangi, Wardha, 442001, India, +91 8237719188

**Keywords:** chronic periodontitis, oxygen-releasing gel, antimicrobial properties, wound healing, tissue regeneration, blue m gel, scaling and root planing

## Abstract

**Background:**

Gingival recession and periodontal pockets are the results of the gradual deterioration of the alveolar bone and periodontal ligament caused by periodontitis, which is caused by inflammation of the tissues supporting the tooth. The primary goal of periodontal therapy is to eradicate these aberrant traits. Despite being a popular treatment, scaling and root planing (SRP) has limitations, such as difficulty accessing deeper pockets and root concavities.

**Objective:**

The aim of this study is to assess the effectiveness of oxygen-releasing gel as a supplement to nonsurgical treatment for chronic periodontitis.

**Methods:**

Twenty-two systemically healthy people with sites of chronic periodontitis were the participants of a split-mouth randomized controlled experiment. For every patient, two locations were chosen and divided into two groups at random. In total, 44 sites were chosen and divided into two groups: Group I (the test group) consisted of 22 test sites, while Group II (the control group) consisted of 22 control sites. While group II underwent SRP alone, group I underwent SRP followed by the use of BlueM oral gel. Plaque index, full mouth bleeding score, clinical attachment level, and probing pocket depth were measured at baseline, one month, and three months, and were compared appropriately.

**Results:**

All the clinical measurements will be recorded. It can be anticipated that SRP followed by the use of BlueM oral gel will provide additional benefits in the management of chronic periodontitis by promoting better healing and clinical outcomes.

**Conclusions:**

Within the constraints of this study, it can be anticipated that by encouraging improved healing and clinical results in chronic periodontitis, oxygen-releasing gel is more advantageous when used in conjunction with SRP than when SRP is done alone.

## Introduction

### Background

The hallmark of Stage II Grade A periodontitis, by Panos N. Papapanou et al, a chronic multifactorial inflammatory disease associated with dysbiotic plaque biofilms, is the progressive deterioration of the tooth-supporting framework. Its primary features include gingival bleeding, periodontal pocketing, loss of alveolar bone as seen on radiography, and the loss of periodontal tissue support, as seen by clinical attachment level (CAL) [1]. Removing bacteria and halting the disease’s progression are two primary goals of periodontal therapy. Scaling and root planing (SRP) and other conventional nonsurgical procedures aim to permanently disrupt the biofilm and its maintenance [2]. As it is difficult to lower the bacterial burden because of the complex structure of the root and the location of the deposits, locally injected antimicrobial medications are used either alone or in conjunction with conventional therapy to target the areas of localized periodontal degeneration [3]. It has been recognized as a means of reducing the emergence of resistance, allergic responses, and systemic issues in contrast to systemic antibiotics [4]. At least 700 bacterial species have been found in the oral cavity, making it one of the most complex and varied human microbiomes. A number of these microorganisms are crucial for preserving the health and regular operation of the mouth. Oral conditions like caries, gingivitis, and periodontal disease can result from changes in the ecology of the oral microbiota [5]. Pathogenic bacteria like Capnocytophaga, Leptotrichia, and Fusobacterium can flourish in the anoxic conditions at the biofilm center, whereas oxidative species like Haemophilus, Aggregatibacter, and Neisseriaceae inhabit the oxygen-rich periphery [6]. Gram-negative bacteria, including *Porphyromonas gingivalis*, *Tannerella forsythia*, and *Treponema denticola*, can control the subgingival biofilm in anoxic periodontal pockets due to interactions between proteins and lectins and carbohydrates. Bacterial cells must communicate and signal one another to form biofilms [6]. The immune system and periodontal pathogens interact intricately to define the activity of periodontal disease. The onset and progression of periodontitis have long been linked to changes in the subgingival microbiome. Changes in the polymicrobial population cause disruptions in host homeostasis in vulnerable individuals, and a number of bacteria are commonly linked to periodontal diseases [7]. In the subgingival region, gram-negative anaerobic bacteria interact with the host’s inflammatory responses to create a hypoxic or reduced oxygen environment. Therefore, oxygen is a necessary molecule for life. A variety of physiological or pathological conditions are linked to variations in tissue oxygen requirements [8]. Hypoxia triggers a local defense or protective cellular responses in a chronic inflammatory illness. Such hypoxic reactions could be the pathophysiology of inflammation, which contributes to the pathogenesis and progression of the disease, if the cause of inflammation cannot be eliminated [9]. In the Netherlands, a group of dental surgeons under the direction of Dr. Peter Blijdorp created a product that relied on the production of active oxygen [10]. By increasing the oxygen content at the wound site, this oxygen-releasing gel helps to speed up the healing process. Additionally, this high concentration of active oxygen aids in wound healing, bleeding gums, and pocket depth reduction. Using this special mixture lowers the risk of irritation and infections while also improving a person’s dental hygiene. Oxygen is essential for many wound healing processes, including collagen synthesis, bacterial oxidative death, re-epithelialization, and angiogenesis [11]. In order to encourage wound healing, topical and hyperbaric oxygen have both been studied as therapeutic approaches. Therefore, the primary goals of topical oxygen therapy using active oxygen-releasing gel are to promote wound healing and avoid dysbiosis [12]. The purpose of this study is to evaluate how well oxygen-releasing gel works as a local medication to treat chronic periodontitis [13].

### Objectives

1. To compare the reduction in probing depth following scaling and root planing (SRP) with and without the application of oxygen-releasing gel in the treatment of chronic periodontitis
2. To evaluate the gain in clinical attachment level after SRP with and without the adjunctive use of oxygen-releasing gel
3. To assess the reduction in Full Mouth Bleeding Score (FMBS) as an indicator of improved periodontal health following SRP with and without the oxygen-releasing gel

## Methods

### Overview

This study aims to compare the clinical and microbiological outcomes of SRP with and without the adjunctive use of an oxygen-releasing gel in the treatment of chronic periodontitis.

### Recruitment

Participants in this clinical trial will be those receiving treatment at the Department of Periodontics and Implantology of Sharad Pawar Dental College, Datta Meghe Institute of Higher Education and Research, or those referred for treatment at this institute, who have periodontal pockets larger than or equal to 4 mm in at least ten permanent teeth. All clinical procedures will be performed here. It is thought that this will enhance trial adherence and recruitment because they will already be receiving treatment.

#### Inclusion Criteria

Participants eligible for the study were motivated to undergo therapy and complete follow-up visits. Individuals who provided written informed consent were included. Participants were required to have a minimum of 15 natural teeth and be in good physical and systemic health. Only patients with clinical attachment loss ≥1 mm and diagnosed with chronic generalized periodontitis, presenting with at least 2–3 sites exhibiting periodontal pocket depths ≥4 mm, were selected for the study.

#### Exclusion Criteria

Patients who had undergone periodontal therapy within the previous 6 months or had received antibiotic therapy during the preceding 3 months were excluded. Individuals who were pregnant or lactating, those with known allergies to the study medications, and regular users of mouthwash were also excluded. Additionally, chronic alcohol users were not included in the study.

### Sample size estimations

The sample size was calculated using the formula for comparison of two independent means:


$$\begin{array}{l} n1=(\sigma 12+\sigma 22/\kappa )(Z1-\alpha /2+Z1-\beta )2\Delta 2n_{1}\\ =\frac{(\sigma_{1}^{2}+\sigma_{2}^{2}/\kappa )(Z_{1-\alpha /2}+Z_{1-\beta }{)}^{2}}{\Delta^{2}}n1\\ =\Delta 2(\sigma 12+\sigma 22/\kappa )(Z1-\alpha /2+Z1-\beta )2 \end{array}$$

where n₁ and n₂ represent the sample sizes of groups 1 and 2, respectively; σ₁ and σ₂ denote the standard deviations of groups 1 and 2; Δ represents the expected difference in group means; κ is the allocation ratio (n₂/n₁); Z₁-α/2 corresponds to the standard normal deviate for a two-sided significance level (1.96 for a 95% confidence interval); and Z₁-β represents the standard normal deviate corresponding to the study power (0.84 for 80% power).

Based on previous findings reported by Anuradha et al [14], the mean Gingival Index was 0.52 in group I and 0.88 in group II, with standard deviations of 0.51 and 0.33, respectively. The expected mean difference (Δ) was therefore calculated as 0.36 (0.88 − 0.52). Assuming an allocation ratio (κ) of 1, a power of 80%, and a significance level of 5% (two-sided), the calculated sample size was 22 participants per group.

### Randomization

44 participants each will receive a different treatment as allocated to Groups 1 and 2 after scaling and root planing are completed. For the participant referring to Group I, oxygen-releasing gel will be administered locally, and for Group II, the supra and subgingival areas will be treated with SRP therapy alone. The flip coin approach will be used for randomization in order to distribute participants into Groups I and II. A separate researcher who is not involved in the methods will create the allocation sequence.

### Procedure

Participants will be enrolled and randomly assigned to interventions by the researchers in charge of the therapy application. Researchers will undergo training before data collection and evaluations, following the procedures and outcome measures outlined. The UNC-15 probe, which measures plaque index (PI), FMBS, Probing Pocket Depth (PPD), and CAL, will be used to evaluate participants’ periodontal clinical characteristics. Every patient will get instructions on the proper brushing technique as well as a demonstration of good oral hygiene practices.

At the first appointment, each patient will receive full-mouth supragingival scaling. After 1 week, on the second appointment, under local anesthesia of 2% lidocaine with 1:100,000 epinephrine, subgingival root planing will be done using Gracey curettes (Hu-Friedy). Following subgingival SRP, the experimental site will be segregated for patients in Group I. Oxygen-releasing gel will be administered in all sites with a probing depth of ≥4 mm over the course of five to ten minutes [7].

Regarding patients in Group II, they will be treated with subgingival SRP alone.

### Statistical Analysis

The calculation of mean and SD will be done, and the mean data will be analyzed using a standard statistical method to determine statistical significance. Examiners performing outcome assessments are blinded to group allocation. The study includes assessor blinding. Outcome assessors will be unaware of participant group allocation to reduce potential bias. All assessments will be conducted using standardized tools to maintain consistency and objectivity. Data from the baseline, one month, and three months for each group will be evaluated using descriptive and inferential statistics, including the *χ*^2^ test, Student paired and unpaired t test, one-way ANOVA, and Tukey test. The analysis will be conducted using SPSS (version 27.0; IBM Corp) and GraphPad Prism (version 7.0) software, with a significance threshold of *P*<.05. A *P* value below 0.05 will be regarded as significant, but a *P* value above 0.05 will be regarded as not statistically significant.

### Evaluation Outcomes

The primary outcome of this study is to measure probing pocket depth, and secondary outcomes will be PI, FMBS, and CAL.

Secondary outcomes would complement the primary clinical measurements, providing a more comprehensive understanding of how the oxygen-releasing gel contributes to the overall treatment of chronic periodontitis when added to SRP.

### Ethical Considerations

Ethics committee approval was obtained for this study (Ref No. DMIHER(DU)/IEC/2025/507) from the Datta Meghe Institute of Higher Education & Research (Deemed To Be University). A written informed consent form approved by the university’s institutional ethics committee must be read, comprehended, and signed by research participants. The consent form will be obtained by the same researcher, a dentist, who is in charge of the treatments. The researcher will go over all the steps that need to be taken, the trial’s goal, the advantages and disadvantages of taking part in the study, and the fact that volunteers are free to decide whether or not to continue with the study at any point without facing any consequences. Participants will be provided with an informed consent form, clearly describing the study’s aim, procedures, risks, benefits, and their right to opt out at any time. Consent for publication of data will be obtained and will be used for all analyses, including secondary analyses. We will confirm that the original consent and institutional review board approval explicitly cover the use of primary and secondary data. Collection of data and entering the data into the database for screening and randomization will be done by the primary investigator. The privacy of prospective and enrolled participants’ personal information will be upheld to ensure confidentiality before, during, and after the trial. We ensure that the data collected will be fully anonymized, with no personally identifiable information retained or linked to any participant. As there are no interventions involved and as only a cross-sectional survey will be conducted, in which the data will be collected via an interview and anthropometric measurements, participants will not be provided any compensation in this study.

## Results

The study was not funded. Recruitment began in April 2025 and is ongoing. As of January 2026, 22 participants have been enrolled. Data collection is ongoing, and statistical analysis has not yet been completed. The results are expected to be published in Winter 2026. All the clinical measurements will be recorded at the baseline, 1 month, and 3 months. PIFMBS, PPD, and CAL will be used to assess the patient’s dental hygiene state. Oxygen-releasing gel may exhibit a higher reduction in clinical parameters.

## Discussion

### Principal Findings

In recent years, adjunctive therapies have been explored to enhance the effects of SRP. One promising approach involves the use of oxygen-releasing gels, which have been shown to possess antimicrobial and tissue-healing properties. These gels release oxygen upon contact with moisture in the periodontal pocket, creating an unfavorable environment for anaerobic bacteria while promoting oxygenation of the periodontal tissues, which may aid in healing and tissue regeneration. The increased oxygen concentration may also help reduce the inflammatory response and facilitate faster wound healing.

A new oxygen-rich mixture called BlueM gel was developed by a team of dentists in the Netherlands. Its main components, sodium perborate and honey enzymes, have combined to create amazing results. This composition is unique in its ability to provide wounded tissues with therapeutically high oxygen concentrations. Along with its oxygen-releasing properties, BlueM gel contains compounds that have antibacterial properties. Its strong bactericidal and bacteriostatic qualities, along with its well-known gradual release characteristic—that is, substantivity within the oral cavity—are what give it its effectiveness [14]. Han et al claim that this gel promotes neovascularization, speeds up the production of new blood cells, and increases stem cell synthesis to produce new fibroblasts [15]. Aqua, alcohol, glycerine, silica, sodium saccharin, sodium perborate, citric acid, PEG-32, sodium gluconate, lactoferrin, xanthan gum, and cellulose gum are the components of the BlueM oral gel recipe, which was developed under Peter Blijdrop’s direction. Each of these chemicals has a specific purpose. BlueM products use oxygen as an active ingredient. Studies show that toothpaste with lactoferrin and active oxygen has similar antiplaque and antigingivitic properties as toothpaste with triclosan. Furthermore, the application of oxygen is essential for a number of healing processes, such as collagen synthesis, angiogenesis, and re-epithelialization [16-18]. Molecular oxygen, one of the wound’s most vital nutrients, is necessary for the healing process [19]. For collagen to be released from cells during the reparative process, oxygen acts as a substrate for the hydroxylation of proline and lysine during collagen synthesis [20]. Oxygen also affects the rates of epithelialization and angiogenesis. When oxygen is given at 1‐2 atmospheres absolute, the oxygen supply regulates the rate of epithelialization in both ischemic and normal wounds [10]. An oxygen gradient stimulates angiogenesis; high arterial PO2~ drives angiogenesis toward areas with low oxygen levels [21]. Molecular oxygen is necessary for leukocytes’ oxidative bacterial killing processes. Bacterial membranes are destroyed by the production of free radicals originating from oxygen. Local oxygen tension is intimately correlated with the rate at which harmful radicals are produced and the capacity to kill through oxidative processes [22]. Investigating topical oxygen treatment may help to lessen wound inflammation and hasten the healing process. According to research, topically administering oxygen promotes the generation of reactive oxygen species (ROS), which are directly related to oxygen levels. Because it may readily pass through cellular membranes, hydrogen peroxide (H2O2) is one of the most prevalent and stable ROS. In tiny quantities, it acts as an intracellular messenger. This property of H2O2 promotes collagen production, stimulates the expression of growth factors such asVEGF (vascular endothelial growth factor) and EGF (epidermal growth factor), controls TGF-β signaling (transforming growth factor–beta), and facilitates neutrophil movement [23]. This study’s findings were comparable to those of a survey by Anuradha et al [14], which compared the effects of curcumin gel as an adjuvant to SRP (Test group) and SRP (Control group). Additionally, Doungudomdacha et al [24] showed statistically insignificant changes in PI and GI (gingival index) in both groups when they compared patients with periodontitis with those with periodontally healthy locations. On the other hand, Koul et al’s survey [10] had different findings that mean difference in GI was greater in the test group than in the control group, even though there was no statistically significant difference in PI between the control and test groups when compared intergroup.

Oxygen-releasing gels provide a slow and controlled release of molecular oxygen directly to the affected area, promoting healing and inhibiting anaerobic bacteria with minimal tissue irritation. In contrast, ozone therapy uses ozone gas (O₃), a highly reactive form of oxygen, which rapidly kills microbes but carries risks of toxicity if not carefully handled [25]. Hydrogen peroxide irrigation works by rapidly releasing oxygen and reactive species upon contact with tissues, which can help clean wounds but may damage healthy cells if used excessively. Unlike ozone and H2O2, oxygen-releasing gels offer a sustained, gentler oxygen supply, making them safer for long-term or repeated use [26].

### Strengths of This Study

A randomized controlled trial design, which is regarded as the gold standard for clinical research, is used in this investigation. Dividing individuals into the experimental and control groups at random lowers biases and improves the reproducibility of the findings. Each participant can function as their control thanks to the split-mouth design. As each patient receives both the normal treatment (SRP) and the experimental treatment (oxygen-releasing gel) on separate sides of their mouth, this reduces interpatient variability. Measurable clinical outcomes that are objective indicators of periodontal health and treatment efficacy, like the PI, FMBS, PPD, and CAL, are probably used in this study. This makes it possible to evaluate the oxygen-releasing gel’s effectiveness with clarity.

### Limitations

Although a split-mouth design minimizes patient variability, clinical assessment of outcomes, such as probing depth or attachment level, can be prone to bias. The study may be limited in terms of its generalizability to all patients with chronic periodontitis. Factors such as age, severity of the disease, and comorbidities might influence the applicability of the results to other populations or settings. If there are other factors influencing the outcome (eg, smoking, systemic health conditions), the results could be confounded.

### Conclusion

It can be anticipated that oxygen-releasing gel has the potential to be employed as a safe and effective adjunct to nonsurgical periodontal therapy for the treatment of chronic periodontitis.

## Abbreviations

CAL: clinical attachment level
FMBS: full mouth bleeding score
GI: gingival index
H202: hydrogen peroxide
PI: plaque index
PPD: probing pocket depth
ROS: reactive oxygen species
SRP: scaling and root planing
